# Evaluation of Paralytic Shellfish Toxins in Marine Oyster Farming and Microalgae in the Atlantic Amazon Evidences Safety but Highlights Potential Risks of Shellfish Poisoning

**DOI:** 10.3390/toxins14100654

**Published:** 2022-09-22

**Authors:** Francisco Arimatéia dos Santos Alves, Eliane Brabo de Sousa, Maíra Pompeu Martins, Cássia Christina da Silva Rocha, Silvia Maria Mathes Faustino, Rosivaldo Alcântara Mendes, Marcelo de Oliveira Lima, Maria Paula Cruz Schneider

**Affiliations:** 1 Laboratory of Genomics and Biotechnology, Biological Sciences Institute, Federal University of Pará, Augusto Correa 01, Belém 66075-110, PA, Brazil; 2 Seção de Meio Ambiente, Laboratório de Análise de Resíduos Orgânicos, Instituto Evandro Chagas/SVC/MS, Rod. Br. 316, Km 7, Ananindeua 67030-000, PA, Brazil; 3 Seção de Meio Ambiente, Laboratório de Cianobactérias e Bioindicadores Aquáticos, Instituto Evandro Chagas/SVC/MS, Rod. Br. 316, Km 7, Ananindeua 67030-000, PA, Brazil; 4 Algae Cultivation Laboratory, Federal University of Amapá, Rod. JK, km 02, Macapá 68902-280, AP, Brazil; 5 Seção de Meio Ambiente, Laboratório de Metais e Ecotoxicologia, Instituto Evandro Chagas/SVC/MS, Rod. Br. 316, Km 7, Ananindeua 67030-000, PA, Brazil

**Keywords:** dinoflagellates, paralytic shellfish poison, gonyautoxin, saxitoxin

## Abstract

Marine phycotoxins are organic compounds synthesized by some species of microalgae, which accumulate in the tissues of filter-feeder organisms such as bivalve mollusks. These toxins can cause acute intoxication episodes in humans, a severe threat to aquaculture and fisheries. In the State of Pará, Brazil, oyster farming has community, artisanal and sustainable bases, using mangroves as cultivation environment and seed banks. In small-scale production, there are often no established methods of safeguarding the health of consumers elevating the potential risks of shellfish poisoning outbreaks. Our study evaluated the presence of phycotoxins in oysters cultivated in five municipalities in the region of the Atlantic Amazon (Pará, Brazil) assessing the quality of the final product. We further evaluated the microalgae, water quality, and the spatio-temporal variation of physicochemical factors in the same area. Diatoms dominated the microalgae composition, followed by dinoflagellates, some of which are reported to be potentially toxic and producers of paralytic shellfish toxins. For the first time, we describe the occurrence of the potentially toxic dinoflagellate *Ostreopsis* sp. in the Amazon region. Furthermore, for the first time, toxins were detected in oyster farming in the northeast of the State of Pará, namely GTX2,3, STX, and dc-STX nevertheless, with nontoxic values. The identified toxins represent a potential threat to shellfish consumers.

## 1. Introduction

Marine phycotoxins are organic compounds produced by unicellular microalgae (Chromista), mostly by dinoflagellates and diatoms [1]. Among dinoflagellates, the planktonic species *Alexandrium*, *Dinophysis*, *Gymnodinium*, and benthic species *Prorocentrum*, and *Ostreopsis,* are the most reported toxin producers [2,3,4,5,6]. The genus *Pseudo-nitzschia* are the main planktonic diatoms involved in these toxic events [7]. Although cyanobacteria, also called blue-green algae, are true bacteria, they are likewise responsible for producing a wide range of phycotoxins in marine environments [8,9].

Outbreaks of intoxication in humans due to marine biotoxins occur mainly through consuming contaminated shellfish. Despite their high nutritional value, marine shellfish have caused concern about the health quality of products as it is a suitable substrate for the proliferation of pathogenic microorganisms and bioaccumulating toxic substances in their tissues. The five major human toxic syndromes associated with algal toxins poisoning are amnesic shellfish poisoning (ASP), diarrhoetic shellfish poisoning (DSP), neurotoxic shellfish poisoning (NSP), azaspiracid shellfish poisoning (AZP), and paralytic shellfish poisoning (PSP) [10].

PSP is one of the most studied intoxications, causing severe symptoms in humans. The associated paralytic shellfish toxins (PSTs) are categorized according to the structural characteristics of their functional groups. In decreasing order of toxicity, they are divided into carbamoyl toxins (saxitoxin—STX, neosaxitoxin—NEO, and gonyautoxins—GTX1 to GTX4); decarbamoyl derivatives (dcGTX1 to dcGTX4, dcSTX and dcNEO), and N-sulforcarbamoyl derivatives (four C–toxins (C1–4), gonyautoxin–5 (GTX5, formerly B1) and gonyautoxin–6 (GTX–6), formerly B2 [11,12]. Due to the potential impacts caused by PSTs-contaminated seafood consumption on marine wildlife and human health, the detection, and control of such toxins are required [13].

The monitoring of shellfish growing regions for changes in water quality is carried out as a food safety measure in many countries which have established limits on the consumption of shellfish contaminated by phycotoxins [14,15]. In Brazil, a rapid expansion of oyster cultivation in coastal regions’ communities highlights the importance of monitoring water quality and the associated risk of accumulating these marine toxins in shellfish [16]. Studies on the retention of microalgae phycotoxins in bivalve mollusks are scarce, recent, and concentrated in the southern regions of the country where most of the shellfish marine farms are located, comprising 97.9% of the national oyster and oyster seeds production [17,18,19]. Santa Catarina is the only Brazilian State that monitors bivalve cultivation areas [17,20].

The Brazilian Amazon coast occupies a coastal strip between the Oiapoque River at Amapá State (5° N, 51° W) and the São Marcos Bay at Maranhão State (2° S, 44° W). Meteorological and oceanographic features are marked by high annual precipitation (up to 3300 mm), high temperatures (>20 °C) with low annual thermal variation, wide continental shelf (~330 km), macrotidal regime (ranging from 6 m to 12 m), extensive area of mangroves, fluvial discharge from dozens of estuaries, including that of the Amazon river, high sediment accretions, and high input of nutrients and organic matter [21,22]. The Amazon Region contains the largest river basin in the world, the Amazon River, which contributes to approximately 16% of freshwater discharged into the oceans [23]. This region includes the coast of Pará State, with extensive areas of mangroves, where oyster farming has community, artisanal and sustainable bases, using the mangroves as a cultivation environment and seed bank [24]. The State of Pará is increasing the production of oysters, and it has the potential to expand production even further, considering the extension and relative preservation of the mangrove coast and the low impact of urban and industrial development [25,26]. The main cultivated and commercialized species is *Crassostrea gasar* (Adanson, 1757), harvested from their natural environment [24].

Although *C. gasar* is farmed in small-scale production, since in the northern coast of Brazil the activity is growing both in production and technological improvement, meeting the sanitary quality standards of food safety surveillance agencies is crucial [24,27,28]. Periodic monitoring of the cultured oysters and water quality evaluating the bioaccumulation of toxins in farming products is fundamental to ensure safe seafood production for human consumption. Thus, the objective of this study was to assess the water quality and the spatio-temporal variation of physicochemical factors and microalgae in the oyster farming region of the Atlantic Amazon in Pará State (Brazil), monitoring the presence of phycotoxins in oysters cultivated on a small scale in the region known as the “Salgado Paraense”. Although in reduced concentrations, toxins were identified, presenting a potential threat to shellfish consumers.

## 2. Results

### 2.1. Physicochemical Characteristics of Water

The multivariate analysis of variance showed differences between months, rainy and dry seasons of the present study. The dry period presented high depth, transparency, salinity, alkalinity, hardness, BOD (biochemical oxygen demand), COD (chemical oxygen demand), sulfate, nitrite, and nitrate than other months. The latter two parameters were increased in the dry season compared to the rainy season. On the other hand, in March (rainy season), an opposite pattern for these parameters was observed compared to the other evaluated months (Supplemental Table S1).

A singular pattern was observed in the municipality Augusto Corrêa, compared to the other sample points. The waters had higher salinity, alkalinity, hardness, BOD, COD, apparent color, total suspended solids, and total dissolved solids. The water quality was altered in terms of dissolved oxygen (less than 5 mg/L, according to the limit stipulated by Brazilian legislation for brackish water), nitrate (up to 0.40 mg/L), and nitrite showed the highest values (0.08 ± 0.18 mg/L).

Dissolved oxygen was also altered in November (2.7 ± 1.7 mg/L) in the Salinas sample, with an average of 4.86 mg/L. In every evaluated month, seasonal period, depth of Euphotic Zone (EuZ), and oyster farming location, nitrate levels were above the limit stipulated by Brazilian legislation for brackish water. Nitrate concentration is a critical parameter for water quality (the limit is up to 0.040 mg/L, in accordance with Brazilian legislation) reaching average values of 39 mg/L in the dry season, 49 mg/L on the water surface, and 72 mg/L in the oyster farming in Augusto Corrêa.

### 2.2. Microalgae

**Figure 1:**
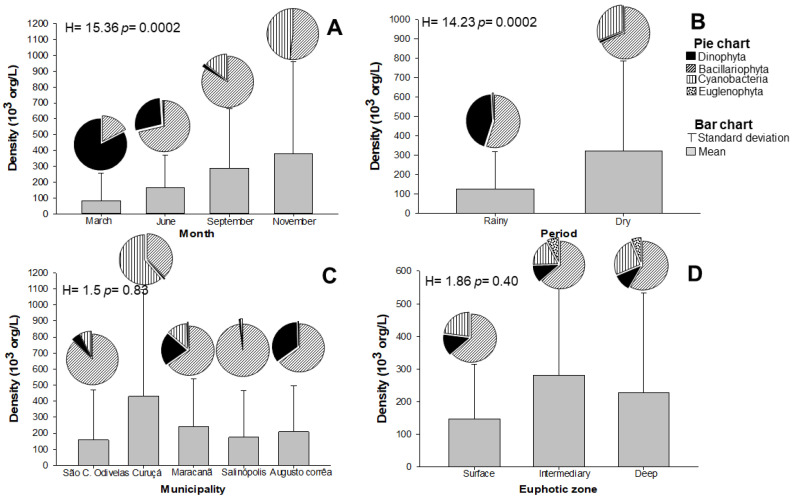
Spatial and temporal variation of the average and standard deviation of microalgae density in the oyster farming region of the State of Pará (Brazil) by (**A**) Month; (**B**) seasonal period; (**C**) location, and (**D**) Euphotic Zone (EuZ) depth. The values represent the average per proposed parameter and at each quadrant, we present individually and independently analyzed data.

A total of 102 species distributed in four microalgae divisions were identified: diatoms (Bacillariophyta) were the most representative algae group, with 84 species, followed by dinoflagellates (Dinophyta) with 11 species. The sample collected in March (rainy season) presented the lowest density (82.56 ± 173.7 × 10^3^ org/L), while in November, the highest density was observed (377.2 ± 584.7 × 10^3^ org/L) (Figure 1A). The overall evaluation of samples collected in dry and rainy periods revealed higher density in the dry months (323.3 ± 463.4 × 10^3^ org/L) than in the rainy months (Figure 1B).

Diatoms were predominantly identified in June (rainy season), September (dry month), and November (dry month) (Figure 1A). In March (rainy season), dinoflagellates were more representative, comprising 76% of the sample composition. The representativeness of cyanobacteria species increased from September to November (dry period), corresponding to 30% and 46% of the microalgae, respectively (Figure 1A). There was no significant difference in density among oyster farming municipalities (H = 1.5; *p* = 0.83) and depths of the EuZ (H = 1.86; *p* = 0.40) (Figure 1C, D). Elevated representativeness of cyanobacteria was observed in Curuçá (72%). The standard deviation reflects the high number of less abundant species (with low densities) counterbalancing the other few species that were more representative (more abundant).

### 2.3. Redundancy Analysis

**Figure 2:**
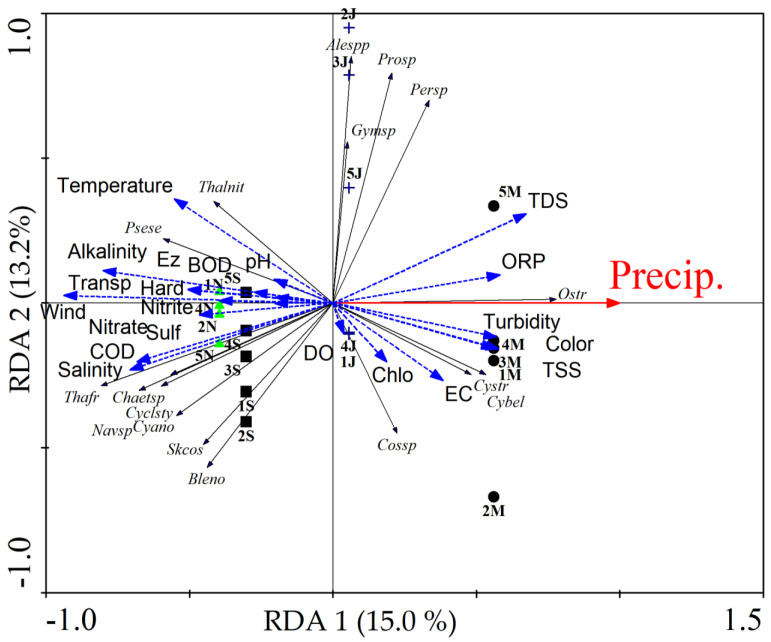
Redundancy analysis (RDA) between spatio-temporal distribution of microalgae and environmental factors in the oyster farming region of the State of Pará (Brazil): Precip.—precipitation; TDS—total dissolved solids; ORP—oxidation-reduction potential; Color—apparent color; TDS—total dissolved solids; TSS—total suspended solids; Ez—euphotic zone; Wind—wind speed; Transp—transparency; Sulf—Sulfate; Hard—Hardness; DO—dissolved oxygen; COD—chemical oxygen demand; BOD—biochemical oxygen demand; EC—electrical conductivity; Chlo—chlorophyll-*a*; *Alespp*—*Alexandrium* spp.; *Gymsp*—*Gymnodinium* sp.; *Prosp*—*Prorocentrum* sp.; *Persp*—*Peridinium* sp.; *Ostr*—*Ostreopsis* sp.; *Cystr*—*Cyclotella striata*; *Cybel*—*Cymatosira belgica*; *Cossp*—*Coscinodiscus* sp.; *Bleno*—*Bleakeleya notate; Skcos*—*Skeletonema costatum*; *Cyano*—*Filamentous cyanobacteria*; *Navsp*—*Navicula* sp.; *Cyclsty*—*Cyclotella stylorum*; *Chaetsp*—*Chaetoceros* sp.; *Thafr*—*Thalassionema frauenfeldii*; *Psese*—*Pseudonitzchia seriata*; *Thalnit*—*Thalassionema nitzschioides*.

The first two axes of the redundancy analysis (RDA) represented 28.2% of the species variation concerning physicochemical and climatic factors. Precipitation was the environmental factor that most influenced species distribution among oyster farming regions (r = 0.938). Axis 1 (15.0%) established a seasonal pattern of the samples, where the March samples (rainy period) were established in the positive quadrant and correlated with the STD variables (r = 0.632) with the species *Ostreopsis* sp., *Cyclotella striata* (Kütz.) Grunow, *Cymatosira belgica* Grunow, and *Trachelomonas volvocina* (Ehrenberg) Ehrenberg as important indicators for the rainy season (IndVal = 50% and *p* = 0.03). On the other hand, the samples from September and November (dry period) were positioned in the negative quadrant (Figure 2). These samples were correlated with wind speed (r = −0.879), transparency (r = 0.500), COD (r = −0.640), and salinity (r = −0.665). The species *Pseudonitzchia seriata* (Cleve) H. Peragallo, *Thalassionema frauenfeldii* (Grunow) Tempère&Peragallo, *Chaetoceros* sp., *Cyclotella stylorum* Brightwell, and filamentous cyanobacteria were statistically significant indicators (*p* < 0.05) of this period with IndVal of 64%, 77.7%, 55.6%, 66.7%, and 58.5%, respectively.

Axis 2 (13.2%) separated June samples from the other months according to the dinoflagellate species *Peridinium* sp., *Alexandrium* spp., *Prorocentrum* sp., and *Gymnodinium* sp. These species were indicators of June with IndVal = 27.6% (*p* = 0.04), IndVal = 26.7% (*p* = 0.011), IndVal = 20.6% (*p* = 0.026), IndVal = 20.0% (*p* = 0.04), respectively. However, there were no environmental variables correlated with this axis, that is, it was not possible to specify the environmental factors that promoted the growth of these species in June.

### 2.4. Identification of PSPs in Oysters by HPLC-FLD

**Figure 3:**
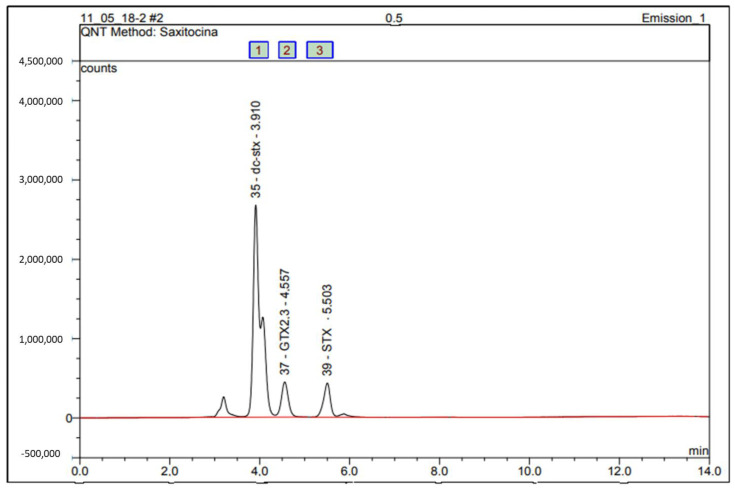
HPLC-FLD chromatogram of PSP toxin standards (dc-STX, GTX2,3 e STX) using pre-column oxidation with periodate. Peak identity: 1—dc-STX; 2—GTX2,3; 3—STX.

The PSP toxin profile was identified by comparing sample chromatograms with certified standards (dc-STX, GTX2,3, STX, NEO, and GTX1.4). The concentration of each PSP toxin was determined using calibration charts with five concentration levels. The GTX2,3, STX, and dc-STX variants were detected in all study regions (Figure 3).

**Figure 4:**
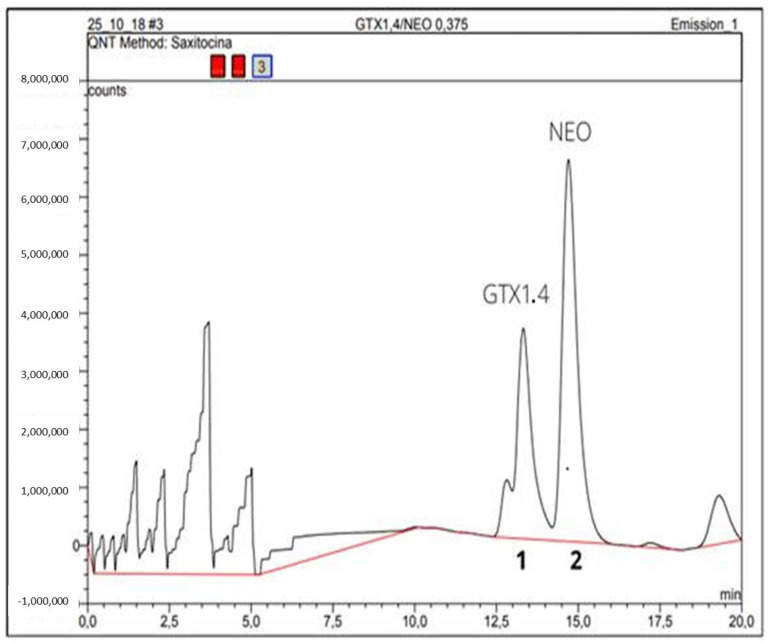
HPLC-FLD chromatogram of PSP toxin standards (GTX-1,4 and NEO) using pre-column oxidation with periodate. Peak identity: 1—GTX1.4; 2—NEO.

PSP values ranged during the evaluated period from 0.00032 to 0.421 mg STX equiv./Kg of oyster flesh. The concentrations of the GTX1,4 and NEO variants (Figure 4) were below the limits of detection (LOD).

In Augusto Corrêa samples, PSP concentrations in oysters ranged from 0.0005 (March) to 0.155mg STX equiv./Kg of oyster flesh (September). In Salinópolis, concentrations ranged from 0.00024 mg STX equiv./Kg (March) to 0.421 mg STX equiv./Kg of oyster flesh (September). In the Curuçá samples, PSP concentrations were below the LOD, with concentrations ranging from 0.0018 (September) to 0.031 mg STX equiv./Kg of oyster flesh (November). Likewise Curuça, in the Maracanã samples, the concentrations were below the LOD and ranged from 0.0003 mg STX equiv./Kg (March) to 0.141 mg STX equiv./Kg of oyster flesh (September). In São Caetano de Odivelas samples, PSP concentrations ranged from 0.0004 to 0.106 mg STX equiv./Kg of oyster flesh in September. In Curuçá, 0.031 mg STX equiv./Kg of oyster flesh of GTX2.3 was detected in November (Table 1).

The detected toxin levels are below the value considered as a risk to human health, established by the National Program for Hygienic-Sanitary Control of Bivalves Mollusks (PNCMB) of the Ministry of Agriculture, Livestock and Food Supply (Ministério da Agricultura, Pecuária e Abastecimento, MAPA). Brazilian regulation follows the recommendations of the European Union, whose maximum acceptable limit for PSP is 80 μg STX eq./100 g of shellfish meat.

## 3. Discussion

Seasonality, especially precipitation, is the main variable that influences the dynamics of microalgae and physicochemical properties of water in the microregion known as “Salgado Paraense”. Precipitation is widely recognized as the most important climatological variable in the tropical region, including the Amazonian coastal region [29], which has the Amazon basin as the main promoter of the hydrological cycle of the entire region [30].

The coastal sector of the Amazon has an average annual total of precipitation that varies around 3400 mm and has the highest rainfall amplitude between the wettest quarter (February to April) and the least rainy (September to November), approximately 560 mm when compared to other regions of the Eastern Amazon [29], confirming the greater importance of rainfall for this region. Rainfall influences the inputs of sediments, organic matter, and salinity in the study region [31]. In the coastal regions of the Amazon, heavy rains during the first six months of the year (average of 326.8 mm) dilute the effects of marine influence on estuarine waters [32]. On the other hand, they increase the influence of limnetic bodies on coastal waters, as in the Tocantins River over the Pará River estuary [33].

Sampaio et al. (2020) studied the oyster farming region of northeastern Pará, the same region where our study was carried out. They found that the evaluated variables changed between the seasonal periods, including salinity, dissolved oxygen, and redox potential, consistent with the present study. The alterations in the physicochemical characteristics of the water seem to be characteristics of the cultivation sites, inserted in mangrove areas. Physicochemical characteristics variations were more noticeable in Augusto Corrêa, which has the largest extension of mangroves and direct contact with the Atlantic Ocean. Despite the strong seasonality, the microplankton fraction of the collected phytoplankton has no significant temporal and spatial variations revealing a favorable environment for these organisms throughout the year. Our results oppose the analysis previously conducted in Bragança, another point in the coastal regions of Pará [32]. The microalgae groups, however, showed expected behavior, such as the representativeness and abundance of diatoms in brackish and saline environments in the world and in the study region [32,34,35,36,37].

Dinoflagellates were the second most abundant group in waters more brackish than saline. The identified species are potentially PSP producers [38]. Among the identified dinoflagellates, we highlight *Ostreopsis* sp., which had a high density (614.7 × 10^3^ org/L) in the least rainy month, compared to the density of the other microalgae in the present study. *Ostreopsis* is a small, benthic, marine dinoflagellate that frequently forms toxic blooms in temperate and tropical waters [39]. There are records of this dinoflagellate in the NE Atlantic, in Portugal, associated with light winds, which allow the formation of bloom in the substrate, and high waves, which resuspend the cells to the surface [40]. In Brazil, this dinoflagellate was identified in the Currais Archipelago in mucilaginous and brownish-colored blooms reaching the order of 10^5^ cells per cm^2^ associated with ovatoxin-a and ovatoxin-b profiles [41]. In our study, *Ostreopsis* was identified at the optical microscopy level. Further analyses are required to confirm if it falls within an already described species and if the species is a toxin producer.

Different species of dinoflagellates produce various PSP analogs, and outbreaks of these toxins previously studied have been mainly attributed to the ingestion of mussels and, less frequently, to other bivalves [42,43]. Mussels are more likely to accumulate toxins for long periods and thus reach higher levels of contamination than other mollusks, representing a vector of transmission to humans. In Santa Catarina State, Brazil, in 2006, a maximum concentration of 600 µg STX equiv./Kg of oyster flesh was found in brown mussels *Perna perna* [44], in areas where high densities of *Gymnodinium catenatum*. The occurrence of PSP in shellfish farming areas has been investigated since 2006 in the State of Santa Catarina. In 2013, PSP toxicity was analyzed in mussels of the *Perna* species in a culture farm in a mariculture area in the city of Arraial do Cabo, State of Rio de Janeiro [45].

The dinoflagellates (Dinophyta) were more representative in the month of June (rainy season) in Maracanã (39.12 ± 52.4 × 10^3^ org/L) and Augusto Corrêa (15.6 ± 33.0 × 10^3^ org/L), and during the analysis of redundancy axis 2 (13.2%) separated the June samples from the other months according to the dinoflagellate species *Peridinium* sp., *Alexandrium* spp., *Prorocentrum* sp., and *Gymnodinium* sp. The presence of dinoflagellates in the wet and dry months of 2017, mainly in samples from June to September, suggests that *Alexandrium* ssp. and *Gymnodinium* sp. may have proliferated over time. Following the density analyses, the maximum values reported were in the month of June (rainy season), and in general, the density values are correlated with the toxicity found in the tissues of the oysters. These facts indicate the possible correlation between PSP toxins and mollusks, and the occurrence of toxic species, in which the presence of PSP toxin seems to coincide with the higher density of *Alexandrium* spp., and *Gymnodinium* sp. identified during the study.

The results of this study are in agreement with long-term statistical studies conducted throughout the world [46]. However, it is not predictable when a bloom of dinoflagellates will develop and neither is the population density of some toxic species. Climatic and environmental conditions such as changes in salinity, rising water temperature, and increased nutrients and sunlight trigger cyst germination to a vegetative stage that allows for rapid reproduction [46].

In Sardinia, *Alexandrium* species were the main causes of toxicity in mollusks. In this study, a discrepancy in abundance was observed, that is, the low number of cells and the high level of toxicity [47,48], similar to the present studies, where the densities of *Alexandrium* spp. (11.4 × 10^3^ org/L), and *Gymnodinium* sp. (10.9 × 10^3^ org/L) were low but sufficient to produce detectable toxins.

The presence of *Alexandrium* species does not imply that PST contamination occurs in mollusks, as variables (sampling period, wind speed, upwelling, and others) can have an important effect [5], mainly in the consumption of these algae by different types of mollusks. For example, mussels are able to accumulate more PSTs and more quickly during algal blooms, unlike scallops, they retain high levels of toxins for much longer and oysters and some clams have low toxicity [49]. This may explain the low concentrations of PSP variants in our study, but these levels may have been much higher in the environment, but the samples collected were only from oysters and not from mussels that are also cultivated in the municipalities, but for community consumption.

There was a difference in the PSP toxin profiles, where GTX2,3 was found in all oyster farming locations, followed by STX and dc-STX. The profiles, NEO and GTX1,4 were not detected. This can be explained, in part, by the methodology applied in the extraction of toxins. The boiling acid 0.1M HCL extraction method converts C3,4 to GTX1,4 and GTX6 (B2) to NEO, causing toxins with an N-sulfocarbamoyl moiety at position 13 to be converted to their carbamoyl counterparts at low pH [50].

Mollusks contaminated by PSP-producing microalgae may present different toxin profiles from the causative organism [51]. This transformation may be due to different facts, such as the chemical properties of toxins, such as epimerization, which occurs spontaneously in toxins. The different profiles could also be due to chemical transformation within the shellfish, since natural reductants commonly found in shellfish, such as glutathione and cysteine, can cause elimination of the N-1 OH group and also the elimination of the O-sulfate Group at position 11 [51], or even by shellfish enzymatic activity, since carbamoylase enzymes catalyze the hydrolysis of the carbamoyl or N-sulfocarbamoyl moiety of PSP toxins, thus turning them into the corresponding carbamoyl analogues.

For the first time, the variants GTX2,3, STX, and dc-STX were detected in the Amazon region, specifically in the coastal area, presenting results of great relevance, which must guide the monitoring programs on water quality and food security in the region. Although the estimated toxin concentrations did not pose risks to the oyster consumer population, our results highlight the necessity of controlling the level of toxins to protect the consumers’ health and avoid damage to the fishing economy due to interdiction of cultivated areas.

## 4. Conclusions

This study presents the first occurrence in the Amazon of the variants GTX2,3, STX, and dc-STX in oyster farming regions in the northeast of the State of Pará in not harmful concentrations to human health. However, our results highlight a potential risk for consumers who demand public agencies’ attention on water quality and food safety. We also emphasize the first occurrence of *Ostreopsis*, a cosmopolitan benthic dinoflagellate associated with toxin production. Changes in water quality in oyster farming regions of the Atlantic Amazon may be associated with the lack of basic sanitation in the municipalities and the biogeochemical cycles of mangroves.

## 5. Materials and Methods

### 5.1. Study Site and Samples

The study area is located on the Brazilian Amazon coast, in the northeast of the State of Pará, in the hydrographic microregion known as “Salgado Paraense”, which comprises 12 municipalities and a population of 320,256 inhabitants. The climate of the region is megathermic type Af, according to the Köppen classification, characterized as a hot-humid tropical climate, with rainfall in all seasons and an annual mean temperature between 24 to 26 °C [52]. The Amazon coastal region has the wettest quarter from February to April and the least rainy period from September to November [29]. The rains are more abundant from December to May, with rainfall amounts usually exceeding 200 mm per month, but often even exceeding 300 mm. In this region, mangroves can extend for more than 40 km towards the land following the course of numerous small estuaries and bays under macrotidal regimes [53].

**Figure 5:**
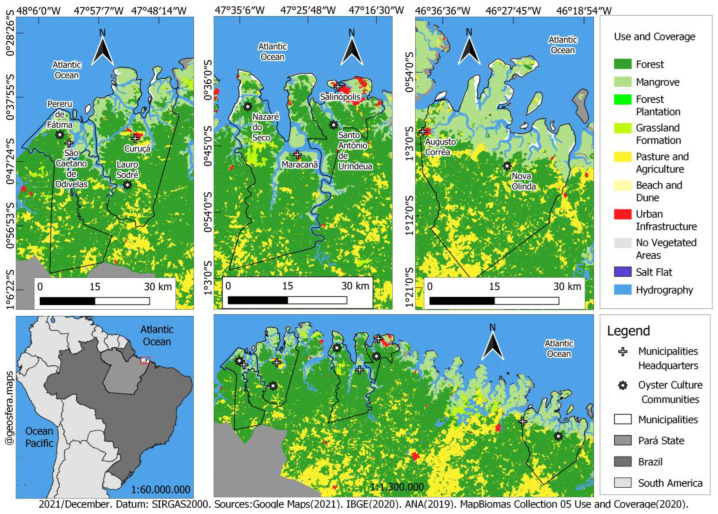
Geographical map of sampling points. Uper image: Northeast coast of Pará divided by municipalities with oyster farming activities (Eastern Amazon, Brazil). The municipalities studied are indicated with a plus sign. From left to right: São Caetano de Odivelas (Pererú de Fátima community), Curuçá (Lauro Sodré community), Maracanã (Nazaré do Seco community), Salinópolis (Santo Antônio de Urindeua), and Augusto Corrêa (Nova Olinda). Bottom image, left: Location of the state of Pará, northern Brazil, eastern Amazonia. Bottom image, right: sampling points are enlarged in the map (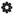 indicates oyster culture communities’ location). Geographic coordinates of the sampling points are provided in the methods section.

The microregion known as “Salgado Paraense” has an average Human Development Index (HDI) of 0.578, an economy predominantly based on agriculture, aquaculture, plant extraction, hunting and fishing, public administration, services, and commerce [20]. The oyster farming occurs in communities in the municipalities of São Caetano de Odivelas (Pererú de Fátima community), Curuçá (Lauro Sodré community), Maracanã (Nazaré do Seco community), Salinópolis (Santo Antônio de Urindeua), and Augusto Corrêa (Nova Olinda) (Figure 5). In these five communities, water samples (aliquots of 1 L) were collected with a Van Dorn bottle at three depths of the Euphotic Zone (EuZ) [54]: near the surface (0.74 ± 0.26 m), intermediate depths (1.1 ± 0.30 m), and deep euphotic zone (2.13 ± 0.6 m). The samples were stored in polypropylene bottles and fixed with lugol acetic [55]. Samples were taken in March, June (rainy months), September, and November 2017 (dry months). *Crassostrea gasar* oysters were collected in the same period, 10 specimens by oyster farming point/month, totaling 200 samples collected during the study period. The oyster’s size ranged from 80 to 99 mm, with a total average weight of 29 g and an average weight of the edible part of 12 g.

### 5.2. Microalgae and Chlorophyll-A

For qualitative microalgae analysis, a 20 μm plankton net was used to drag the water subsurface horizontally for three minutes. The collected material was fixed with 4% formalin solution [55] and analyzed by triocular microscope (Axio Lab. A1, Carl Zeiss, Jena, Germany) with measuring eyepieces (Axiocam MRc, Carl Zeiss, Jena, Germany).

Quantitative microalgae samples were obtained from the water collected using the Van Dorn bottle fixed by lugol acetic. Counting of microalgae using inverse microscopy (Observer D1 Zeiss, Germany) followed the method outlined in APHA 2012, section 10200F [55]. All cells (coenobia, frustules, loric cells, colonies, or filaments) were considered one organism (org/L). Identification, nomenclature, and taxonomic classification were in accordance with the specialized literature [56,57,58,59]. The taxonomic identification of the *Ostreopsis* genus was performed based on its morphological characteristics using light microscopy and, despite its fine teak, contour, teardrop shape, dimensions, golden-brown chloroplasts were the main characteristics observed for this genus [60,61].

The chlorophyll-a samples were vacuum filtered through 0.45µm pore size cellulose filters. The pigments were extracted with 90% acetone and chlorophyll-a were measured according to Standard Methods 10,200 H with a Hach DR6000 spectrophotometer [55].

### 5.3. Physicochemical Characteristics of Water

Water transparency was measured using a Secchi disk. Temperature (°C), the potential of hydrogen (pH), salinity, electrical conductivity (EC), total dissolved solids (TDS), and dissolved oxygen (DO) were measured in situ using a HI 9828 multiparametric probe (HANNA^®^, Smithfield, UT, USA). Parameters were measured of the water samples collected from three depths through the Van Dorn bottle. Analyses followed the Standard Methods for the Examination of Water and Wastewater, section 1060 [55].

Turbidity and apparent color variation were determined by the 2130 B nephelometric method [55] and total suspended solids (TSS) by the photometric method [62]. The biochemical oxygen demand (BOD) was determined by the 5210 B method and the chemical oxygen demand (COD) by the closed reflux colorimetric method (5220D), both determined by UV-VIS spectrometry (DR 3900 model), according to Baird et al. (2012). Nitrite (NO^2−^), nitrate (NO^3−^), hardness, and sulfate (SO_4_^2−^) were determined in an ICS Dual 2000 ion chromatograph (Dionex Corporation, Sunnyvale, CA, USA) with chemical suppression of eluent conductivity, following method 4110 B [55].

The water quality was evaluated according to the conditions and standards of the National Council of the Environment (Conselho Nacional do Meio Ambiente, CONAMA Resolution 357/2005), considering the environment as class I of brackish water (salinity > 0.5‰ to 30‰), since the water can be destined to aquaculture and fishing activity [63]. Precipitation and wind data were provided by the National Institute of Meteorology [64].

### 5.4. Statistical Analysis

The variations of environmental factors were analyzed using a Two-Way Nonparametric Multivariate Analysis of Variance PERMANOVA, since the values even transformed did not reach the multivariate normality through the tests of Mardia and Doornik, and Hansen Omnibus. A Univariate Analysis of Variance was performed using the Kruskal–Wallis tests and Dunn’s post hoc test for density values. For all tests, a statistical significance lower than 5% (*p* < 0.05) was considered. Analyses were performed using the free software Past 4.06 for Windows [65].

The indicator species of the environment (IndVal—Indicative value of the species) were determined as previously described [66]. The statistical significance of IndVal was tested by the Monte Carlo technique through 9,999 permutations [67] using the PCORD 5 software [68]. From the matrix of significantly indicator species, the RDA, canonical ordering analysis was performed using a biological matrix with the significant indicator species (IndVal) transformed into Hellinger and the environmental matrix contains the values of the physicochemical variables, precipitation, and winds, transformed into Rangin [67].

### 5.5. Extraction and Clean-Up Procedure for Shellfish Samples

A total of 80 oysters from the 200 collected samples were opened and their soft tissues were removed from the valves, rinsed with water, and used for quantification of toxins. Extraction and cleaning of oyster samples were performed according to the HPLC pre-column oxidation reference method for PSPs, following AOAC Official Method 2005.06 [50]. Briefly, a 10 g portion of the oyster soft tissue was extracted in 10 mL of 0.1M HCl and boiled for 5 min. The mixture was cooled and centrifuged for 10 min at 4500 rpm; 1 mL of the supernatant was cleaned in a 3 mL SPE C18 cartridge (Supelclean Supelco, Bellefonte, PA, USA) and then diluted to 5 mL with water. The pH was adjusted to 6.0 (± 0.2) with 1% NH_4_OH. The adjusted solution was passed through a 3 mL SPE (COOH) cartridge (Bakerbond, J.T. Baker, USA), and toxins GTX1,4, GTX2.3, and dcGTX2.3 were recovered with 4 mL of 0.01M NaCl solution (fraction 1). The cartridge was then eluted with 5 mL 0.3M NaCl Solution and collected as fraction 2 containing STX, NEO, and dcSTX. The recovered fractions were then analyzed by HPLC-FLD.

### 5.6. HPLC-FLD Analysis

Oxidation products of PSP toxins were obtained [69] and the HPLC-FLD analyses were performed using an Ultimate 3000 Chromatograph with a fluorescence detector (Thermo Fischer Scientific, Waltham, MA, USA). Chromeleon™ 7.2 software (Thermo Fischer Scientific, USA) performed data acquisition and peak integration. Separation was performed on a Supelcosil LC-18 column, 150 × 4.6 mm, 5 μm (Supelco, USA) equipped with a C18 guard column (SecurityGuard™, 4 × 3 mm, Luna, Phenomenex, Torrance, CA, USA). Mobile phase A was made of 0.1M ammonium formate, pH 6 and mobile phase B was made of 0.1M ammonium formate in 5% acetonitrile, pH 6. The column was kept at 32 °C, where 20 μL per sample. Detection wavelengths were set at 340 nm for excitation, 395 nm for quantification, and 430 nm for fluorescence confirmation. The PSP oxidation products were eluted using the following gradient: 100% mobile phase A for the first 5 min, 95% mobile phase A for 5 to 9 min, 70% mobile phase B for 9 to 11 min, 100% mobile phase A in the last 3 min. Before analysis, the pre-column and column were cleaned to avoid or reduce the occurrence of ghost peaks. The procedure consists of elution with deionized water and acetonitrile through the pre-column and column with a flow of 0.2 mL/minute lasting 2 h for each eluent.

Toxins were identified by comparing retention times with wavelength standards and rates. For the quantification of toxins, several matching matrix calculation curves were prepared on oyster extracts (C18 or COOH) using NRC Certified Reference Materials: GTX1,4, STX, dc-STX, GTX2,3, NEO (Institute for Marine Biosciences, National Research Council of Canada, Halifax, NS, Canada). Individual toxin levels were converted to saxitoxin equivalents using the toxicity equivalence factors (TEFs) of STX group toxins proposed by the EFSA panel [70]. The EU Reference Laboratory has adopted this approach for Marine Biotoxins, which recommends their routine use by monitoring laboratories.

## Figures and Tables

**Table 1:** Equivalent concentrations of phycotoxins identified in oyster farming regions of the State of Pará (Amazônia, Brazil). In STX equiv./Kg, Kg refer to oyster flesh.

Sampling Points	Months	GTX1,4 (Mg STX Equiv./Kg)	GTX2,3 (Mg STX Equiv./Kg)	STX (Mg STX Equiv./Kg)	dc-STX (Mg STX Equiv./Kg)	NEO (Mg STX Equiv./Kg)
São Caetano de Odivelas	March	<LOD	<LOD	0.00249	<LOD	<LOD
	June	<LOD	0.0038	0.00052	0.00102	<LOD
	September	<LOD	0.0049	0.106	0.00046	<LOD
	November	<LOD	<LOD	<LOD	<LOD	<LOD
Curuçá	March	<LOD	<LOD	<LOD	<LOD	<LOD
	June	<LOD	<LOD	<LOD	<LOD	<LOD
	September	<LOD	0.0076	<LOD	0.0018	<LOD
	November	<LOD	0.031	<LOD	<LOD	<LOD
Maracanã	March	<LOD	<LOD	<LOD	<LOD	<LOD
	June	<LOD	0.0044	0.000707	0.00273	<LOD
	September	<LOD	0.083	0.141	0.00032	<LOD
	November	<LOD	<LOD	<LOD	<LOD	<LOD
Salinópolis	March	<LOD	<LOD	0.00024	<LOD	<LOD
	June	<LOD	0.003	0.00044	<LOD	<LOD
	September	<LOD	<LOD	0.421	0.001	<LOD
	November	<LOD	<LOD	<LOD	<LOD	<LOD
Augusto Corrêa	March	<LOD	0.018	0.0172	0.00059	<LOD
	June	<LOD	0.012	<LOD	0.0018	<LOD
	September	<LOD	0.023	0.155	0.00103	<LOD
	November	<LOD	<LOD	<LOD	<LOD	<LOD

## Data Availability

Not applicable.

## Supplementary Materials

The following supporting information can be downloaded at: https://www.mdpi.com/article/10.3390/toxins14100654/s1, Table S1: Measurement of the physicochemical parameters and analysis of the water samples from the oyster farming region of the State of Pará, Brazil.
