# Supplementary material for: Evaluation of Paralytic Shellfish Toxins in Marine Oyster Farming and Microalgae in the Atlantic Amazon Evidences Safety but Highlights Potential Risks of Shellfish Poisoning
Source: Toxins (Basel). 2022 Sep 22;14(10):654. doi: 10.3390/toxins14100654 (PMC9611215; doi:10.3390/toxins14100654)
Supplement: Supplementary file 1 [file toxins-14-00654-s001.zip › toxins-1850378-supplementary.pdf]

toxins

MDPI

Article

Evaluation of Paralytic Shellfish Toxins in Marine Oyster Farming and Microalgae in the Atlantic Amazon Evidences Safety but Highlights Potential Risks of Shellfish Poisoning

Francisco Arimatéia dos Santos Alves,Eliane Brabo de Sousa, Maíra Pompeu Martins,
Cássia Christina da Silva Rocha, Silvia Maria Mathes Faustino, Rosivaldo Alcântara Mendes, Marcelo de Oliveira Lima and Maria Paula Cruz Schneider

Table S1: Measurement of the physicochemical parameters and analysis of the water samples from the oyster farming region of the State of Pará, Brazil.

temporal/spatial		ZEU	Transparency	Chlorophyll- a	Temperature	pH	Conductivity	Dissolved oxygen	Dissolved Total Solids	Salinity	ORP	alkalinity	Apparent Color	Toughness	DBO	DQO	Nitrite	Nitrate	STS	Sulfate	turbidity
Month	March	0,4-2,4; (1,1 ± 0,6)	0,4- 0,8; (0,6 ± 0,2)	6,1- 42,7; (14,8 ± 12,0)	26,7- 29,0; (27,9 ± 0,8)	6,5- 10,0; (7,4 ± 0,8)	46,8- 9359,0; (4090,3 ± 2843,4)	4,2- 9,4; (6,0 ± 1,6)	1,2- 8562,0; (2287,9 ± 2748,8)	1,2- 18,6; (6,4 ± 5,7)	67,1- 1515,0; (261,8 ±404,7)	35,0- 110,0; (58,6 ± 25,6)	40,0- 1202,0; (518,5 ± 499,2)	0,8- 4,6; (2,4 ± 1,1)	5,9- 122,0; (20,8 ± 29,5)	14,0- 186,0; (64,1 ±51,8)	0	0,0-2,4; (0,9 ± 0,7)*	2,0- 145,0; (63,5 ± 61,0)	0,2- 5,1; (1,5 ± 2,0)	3,5- 162,0; 67,7 ± 70,0)
	June	0,5- 2,7; (1,2 ± 0,7)	0,5- 0,9; (0,7 ± 0,2)	3,3- 19,6; (9,4 ± 5,7)	28,6- 30,3; (29,6 ± 30,3)	6,7- 8,4; (7,3 ± 0,5)	111,0- 3401,0; (2186,5 ±	2,4- 8,3; (5,0 ± 2,2)	1,5- 7485,0; (1995,3 ±	1,8- 10,6; (5,3 ± 3,5)	50,0- 197,6; (126,5 ± 46,2)	100,0 – 130,0; (106,7 ± 12,3)	57,0- 141,0; (91,3 ± 31,2)	1,1- 161,5; (28,8 ± 55,0)	5,9- 59,0; (38,5 ± 14,9)	19,0 – 585,0; (119,3 ± 139,9)	0,0- 0,1; (0,0 ± 0,0)	0,4- 13,5; (4,8 ± 4,4)*	5,6- 17,5; (9,5 ± 4,2)	0,2- 8,7; (4,6 ± 2,9)	5,0- 24,0; (16,6 – 6,5)
	September	0,6 -3,0; (1,4 ± 0,7)	0,6-1,0; (0,8 ± 0,1)	4,1- 27,4; (11,3 ± 7,4)	28,6- 29,7; (29,3 ± 0,3)	6,9- 8,1; (7,7 ± 0,4)	44,6- 3847,0; (2208,2 ± 1830,7)	0,0 – 14,0; (7,0 ± 4,2)	16,4 – 1923,0; (401,7 ± 786,9)	-2,4- 41,3; (22,7 ± 12,1)	2,1- 65,1; (41,9 ± 24,5)	110,0- 200,0; (162,7 ± 28,5)	23,0- 118,0; (49,7 ± 27,0)	4,0- 269,1; (62,6 ± 65,9)	5,9- 233,0; (42,8 ± 58,5)	1172,0- 2102,0; (1617,7 ± 314,0)	0,0- 0,7; (0,1 ± 0,2)	0,0- 853,2; (70,1 ± 217,8)*	1,0- 9,0; (4,4 ± 2,5)	0,2- 2352,0; (458,3 ± 946,5)	2,0- 37,0; (5,5 ± 8,8)
	November	0,6- 3,3; (1,6 ± 0,9)	0,6- 1,1; (0,9 ± 0,2)	3,3- 23,7; (9,2 ± 7,2)	28,8- 31,1; (29,6 ± 0,8)	6,9- 8,0; (7,5 ± 0,4)	39,3- 54,8; (48,2 ± 6,3)	1,1- 6,3; (2,7 ± 1,7)*	19,6- 27,4; (24,1 ± 3,1)	24,9- 36,1; (30,5 ± 4,7)	14,4- 28,4; (9,9 ± 18,1)	40,0-200,0; (145,8 ± 51,0)	23,0- 95,0; (53,8 ±	0,4- 49,9; (7,4 ± 13,7)	5,9- 233,0; (55,1 ± 62,3)	132,0- 5508,0; (1363,3 ± 1597,2)	0	0	2,0- 11,0; (5,9 ± 3,1)	41,2- 2968,0; (1298,3 ± 1060,7)	2,0- 9,0; (5,8 – 2,6)

temporal/spatial		ZEU	Transparency	Chlorophyll- a	Temperature	pH	Conductivity	Dissolved oxygen	Dissolved Total Solids	Salinity	ORP	alkalinity	Apparent Color	Toughness	DBO	DQO	Nitrite	Nitrate	STS	Sulfate	turbidity
seasonality	Rainy	0,4- 2,7; (1,4 ± 0,63)	0,4- 0,9; (0,62 ± 1,7)	3,3- 42,7; (12,08 ± 9,6)	26,7- 30,3; (28,83 ± 1,1)	6,5- 10,0; (7,4 ± 0,7)	46,8 – 9359,0; (3138,4 ± 2319,3)	2,4- 9,4; (5,5 ± 2,0)	1,2- 8562,0; (2141,6 ± 2607,8)	1,2- 18,6; (5,8 ± 4,7)	50,0- 1515,0; (194,2 ± 291,3)	35- 130; (82,6 ± 31,4)	40,0- 1202,0; (305,0 ± 410, 0)	0,83- 161,5; (15,6 ± 40,5)	5,9- 122,0; (29,7 ± 24,6)	14,0 – 585,0; (91,6 ± 107,4)	0,012- 0,052; (0,023 ± 0,012)	0,006- 13,5; (2,7 ± 3,6)*	2,0- 145,0; (36,5 ± 50,5)	0,17 – 8,7; (3,0 ± 2,9)	3,5- 162,0; (42,1 ± 55,2)
	Dry	0,6- 3,3; (1,5 ± 0,8)	0,6- 1,1; (0,8 ± 0,18)	3,3-27,4; (10,3 ± 7,3)	28,6- 31,1; (29,4 ± 0,6)	7,0- 8,0; (7,6 ± 0,4)	39,3- 3847,0; (1248,2 ± 1732,3)	0,03- 13,9; (5,1 ± 4,0)	16,4 – 1923,0; (233,9 ± 608,2)	2,43- 41,26; (26,16 ± 10,2)	-14,4- 168,5; (84,1 ± 61,2)	40,0- 200,0; (155,2 ± 40,1)	23,0- 118,0; (51,5 ±24,5)	0,42- 269,1; (38,1 ± 56,5)	5,9- 233,0; (48,2 ± 59,3)	132- 5508,0; (1504,6 ± 1072,0)	0,012- 0,663; (0,056 ± 0,124)	0,006- 853,2; (39,0 ± 163,7)*	1,0- 11,0 (5,07 ± 2,8)	0,2 – 2968,0; (831,6 ± 1067,4	2,0- 37,0; (5,6 ± 6,7)
layer Zeu	Surface	0,4- 1,5; (0,74 ± 0,26)	0,4- 1,1; (0,71 ± 0,2)	4,1 -37,6; (10,8 ± 8,4)	26,69- 30,47; (29, 0 ± 9,0)	6,7- 8,3; (7,4 ± 0,46)	39,3- 8156,0; (2102,0 ± 2146,7)	0,03- 14,0; (5,84 ±	1,5- 5494,0; (1026,2 ± 1590,6)	1,8- 41,3; (16,0 ± 14,0)	-9,4- 916,6; (112,0 ± 202,5)	35- 184; (112,0 ± 50,0)	26,5 – 1100; (184,4 ± 318,3)	0,42- 269,1; (30,74 ± 66,4)	5,9- 67; (24,7 ±	24- 2185; (718,0 ± 823,3)	0,01- 0,7; (0,07 ±	0,006- 853,2; (49,0 ± 195,0)*	2,0- 138,5; (21,3 ±	0,1718- 2348,0; (306,2 ±	2,0- 150,0; (26,0 ± 43,3)
	intermediary	0,6- 1,7; (1,1 ± 0,30)		3,3- 42,7; (11,7 ± 10,0)	27,0 – 31,1; (29,1 ± 0,98)	6,5- 9,9; (7,5 ± 0,75)	40,3- 9316; (2310,5 ± 2335,6)	1,1- 9,9; (5,4 ± 2,75)	1,2- 8562,0; (1344,0 ± 2432,7)	1,2- 36,1; (14,5 ± 12,6)	-14,4- 168,5; (84,1 ± 61,2)	35- 200; (118,6 ± 51,6)	30-,0 1202,0; (190,53 ± 335,7)	0,83- 110,0; (24,6 ± 39,5)	6,0- 233,0; (56,0 ± 67,3)	14,0- 5508,0; (867,3 ± 1359,2)	0,01- 0,05; (0,65- 0,014)	0,006 – 10,7; (2,8 ± 3,6)*	2,0- 145,0; (22,0 ± 41,1)	0,17- 2968; (455,2 ± 937,3)	2,0- 162,0; (25,0 3 ± 45,8)
	Deep	1,2- 3,3; (2,13 ± 0,6)		3,3- 30,3; (11,23 ± 7,50)	26,7- 30,9; (29,2 ± 0,95)	6,86- 8,25; (7,5 ± 0,4)	40,6 – 9359,0; (2316,6 ± 2393,1)	1,15- 11,2; (4,7 ± 2,7)*	5,6- 8021,0; (1343,7 ± 2417,8)	2,0- 36,1; (16,0 ± 12,9)	-13,7- 1515,0; (149,7 ± 335,5)	35,0- 200,0 (120,8 ± 53,5)	23,0- 1123,0; (179,6 ± 328,8)	1,3- 161,5; (24,0 ± 40,7)	6,0- 109,0; (34,7 ± 30,0)	19,0- 2102,0; (698,1 ±	0,01- 0,06; (0,028 ±	0,006- 93,0; (8,2 ± 22,0)*	1,0- 137,5; (21,6 ± 40,2)	0,2- 2352,0; (425,3 ± 866,9)	2,0- 154,0; (23,5 3 ± 45,32)
location	São Caetano de Odivelas	0,5- 1,8; (1,0 ± 0,5)	0,5- 0,6; (0,55 ± 0,06)	3,3- 27,4; (10,73 ± 8,3)	26,7- 29,4; (28,4 ± 1,01)	6,8- 10,0; (7,5 ± 0,87)	44,6- 4694,0; (2132,0 ± 1834,6)	1,1- 14,0; (5,8 ± 4,02)	17,9 - 2347,0; (618,3 ± 1041,4)	2,4- 28,9; (16,1 ± 10,8)	14,4- 1515; (179,8 ± 424,2)	35,0- 200,0; (116,2 ± 56,2)	60- 1075; (327,9 ± 442,6)	1,63 - 107,0; (26,1 ± 35,58)	5,9- 46; (25,6 ± 13,2)	24- 5508; (1181,0 ± 1548,4)	0,013- 0,05; (0,02 ± 0,011)	0,006- 93,0; (11,16 ± 27,3)*	4,0 - 131,0; (37,125 ± 55,2)	0,17- 63,7; (16,4 ± 25,4)	3,0 - 147,0; (43,3 ± 61,2)
	Curuçá	0,8- 3; (1,7 ± 0,81)	0,8-1,0; (0,93 ± 0,095)	3,3- 7,8; (6,0 ± 1,52)	27,9- 29,7; (29,0 ± 0,67)	6,7 - 7,4; (7,0 ± 0,17)	39,3- 9359,0; (3601,3 ± 3498,3)	2,2 - 11,61; (6,01 ± 2,7)	1,5- 7485,0; (1565,3 ± 2888,0)	1,8- 26,0; (14,6 ± 9,7)	25,0- 110,0; (63,4 ± 30,5)	43,0- 140,0; (100,2 - 37,5)	25,0- 123,0 (63,6 - 30,37)	0,83- 110; (15,5 ± 31,7)	6,0- 33,0; (18,7 ± 11,5)	26,0- 1284,0; (382 ± 508,0)	0,01- 0,04; (0,015 ± 0,008)	0,006- 14,3; (4,3 ± 5,0)*	1,0- 16,0; (7,6 ± 4,6)	0,2- 1281,0; (300,4 ± 536,2)	2,0- 12,5; (6,3 ± 3,07)

**temporal/spatial**	**ZEU**	**Transparency**	**Chlorophyll- a**	**Temperature**	**pH**	**Conductivity**	**Dissolved oxygen**	**Dissolved Total Solids**	**Salinity**	**ORP**	**alkalinity**	**Apparent Color**	**Toughness**	**DBO**	**DQO**	**Nitrite**	**Nitrate**	**STS**	**Sulfate**	**turbidity**
Maracanã	0,5- 2,4; (1,2 ± 0,63)	0,5- 0,8; (0,63 ± 0,15)	6,8- 42,7; (18,4 ± 14,5)	28,8- 30,3; (29,6 ± 0,57)	7,5- 8,4; (7,8 ± 0,35)	44,6 - 3162,0; (1611,0 ± 1316,3)	4,8- 9,3; (6,89 ± 1,54)	22,3- 8562,0; 8562,0; (2372,4 ± 3429,7)	1,8- 28,9; (13,5 ± 12,0)	64,0- 161,8; (102,9 ± 39,6)	64,0- 180,0; (115,4 ± 50,6)	39,0- 291,0; (131,2 ± 84,3)	1,1 - 65,0; (15,9 ± 23,9)	5,9- 59,0; (31,3 ± 19,44)	19,0- 1854,0; (599,7 ± 818,7)	0,01- 0,116; (0,04 ± 0,032)	0,3- 13,5; (5,24 ± 5,3)*	2,0 - 40,5; (15,83 ± 12,6)	0,17- 5,2; (3,3 ± 2,3)	2,0 - 32,0; (16,2 ± 10,84)
Salinas	0,4- 2,1; (1,05 ± 0,58)	0,4- 0,7; (0,58 ± 0,15)	8,9 - 23,74; (16,42 ± 4,25)	27,5- 31,1; (29,4 ± 1,2)	6,5- 7,62; (7,3 ± 0,34)	51,6- 4267,0; (2202,5 ± 1909,5)	1,2 - 9,31; (4,86 ± 2,26)*	5,5- 5494,0; (1476,5 ± 1613,1)	2,12- 35,53; (10,9 ± 12,9)	11,6- 197,6; (89,0 ± 68,0)	35-180; (110,2 ± 61,52)	39,0- 1202,0; (345,6 ± 483,6)	0,62- 65,0; (12,7 ± 21,3)	5,9- 122,0; (41,5 ± 30,1)	14- ,01854,0; (472,6 ± 736,5)	0,01- 0,12; (0,04 ± 0,03)	0,006- 13,5; (3,62 ± 5,32)*	2,0- 145,0; (41,2 ± 60,0)	0,2- 2968,0; (609,1 ± 1116,5)	2,0- 162,0; (46,8 ± 65,9)
Augusto Corrêa	0,7- 3,3; (1,56 ± 0,82)	0,7-1,1; (0,85 ± 0,17)	3,3- 10,7; (6,5 ± 2,4)	27,9- 30,2; (29,1 ± 0,71)	7,3- 7,9; (7,7 ± 0,3)	54,4- 3401,0; (1510,5 ± 1562,1)	0,03- 9,4; (3,3 ± 2,9)*	1,17- 1701,0; (440,8 ± 757,6)	1,2- 41,3; (22,2 ± 16,5)	2,1- 916,6; (138,1 ± 256,3)	100,0-20,0; (142,75 ± 44,04)	23,0- 66,0; (42,4 ± 16,8)	0,42- 269,1; (58,4 ± 88,6)	5,9- 233,0; (73,5 ± 82,9)	125,0- 2185; (1129,2 ± 1015,4)	0,01- 0,7; (0,08 ± 0,18)*	0,006- 853,2; (72,29 ± 245,9)*	2,0- 9,5; (4,9 ± 2,3)	0,17- 2352,0; (950,5 - 1169,0)	2,0- 37,0; (9,3 ± 10,1)
